# The vascular role of CGRP: a systematic review of human studies

**DOI:** 10.3389/fneur.2023.1204734

**Published:** 2023-07-06

**Authors:** Mohammad Al-Mahdi Al-Karagholi, Veberka Kalatharan, Peter Schunck Fagerberg, Faisal Mohammad Amin

**Affiliations:** Danish Headache Center, Department of Neurology, Rigshospitalet Glostrup, Faculty of Health and Medical Sciences, University of Copenhagen, Copenhagen, Denmark

**Keywords:** Calcitonin gene-related peptide, infusion, hemodynamic, adverse events, headache, migraine

## Abstract

Intravenous infusion of human alpha calcitonin gene-related peptide (h-α-CGRP) has been applied to explore migraine pathogenesis and cerebral hemodynamics during the past three decades. Cumulative data implicate h-α-CGRP in regulating the vascular tone. In this systematic review, we searched PubMed and EMBASE for clinical studies investigating the vascular changes upon intravenous infusion of h-α-CGRP in humans. A total of 386 studies were screened by title and abstract. Of these, 11 studies with 61 healthy participants and 177 participants diagnosed with migraine were included. Several studies reported hemodynamic effects including flushing, palpitation, warm sensation, heart rate (HR), mean arterial blood pressure (MABP), mean blood flow velocity of middle cerebral artery (mean V_MCA_), and diameter of superficial temporal artery (STA). Upon the start of h-α-CGRP infusion, 163 of 165 (99%) participants had flushing, 98 of 155 (63%) participants reported palpitation, and 160 of 165 (97%) participants reported warm sensation. HR increased with 14%–58% and MABP decreased with 7%–12%. The mean V_MCA_ was decreased with 9.5%–21%, and the diameter of the STA was dilated with 41%–43%. The vascular changes lasted from 20 to >120 min. Intravenous infusion of h-α-CGRP caused a universal vasodilation without any serious adverse events. The involvement of CGRP in the systemic hemodynamic raises concerns regarding long-term blockade of CGRP in migraine patients with and without cardiovascular complications.

## 1. Introduction

Calcitonin gene-related peptide (CGRP) is a 37-amino acid vasoactive neuropeptide that belongs to the calcitonin family which includes adrenomedullin, adrenomedullin 2/intermedin and amylin (1). The human CALCA gene codes for the thyroid gland hormone calcitonin and α-CGRP via alternative splicing in neural tissues (2). Upon neuronal stimulation, α-CGRP is released from sensory neurons (mainly C-fibers) via calcium-dependent exocytosis to bind to its receptor located on the cell membrane of several cell types including, smooth muscle cells (3–5), endothelial cells (6), and cardiomyocytes (7). CGRP receptor complex consists of a seven domain G-protein coupled receptor (GPCR) known as calcitonin receptor-like receptor (CRLR) associated to a single transmembrane protein recognized as receptor activity modifying protein-1 (RAMP1). CGRP leads to vasodilation via two distinct mechanisms. Direct activation of the receptor complex in vascular smooth muscle cells causes protein kinase A (PKA)-mediated smooth muscle relaxation, or indirectly by nitric oxide from endothelial cells. CGRP has cardioprotective effects, and several preclinical studies demonstrated its role in cardiovascular health (5, 8–10).

Intravenous infusion of human α-CGRP (h-α-CGRP) has been applied to explore headache and migraine pathogenesis during the past three decades. These studies reported that h-α-CGRP induced headache in healthy individuals and migraine-like attacks in individuals with migraine (11, 12). These findings led to development of drugs that target the CGRP pathway for the acute and preventative treatment of migraine (13). The cardioprotective effects of CGRP (10), including the (1) antihypertensive effects, (2) attenuating cardiac remodeling, and (3) increasing angiogenesis to limit damage associated with the progression of cardiovascular diseases, raises concerns regarding long-term blockade of CGRP in individuals with migraine who are at high risk for ischaemic events or have a history of a myocardial infarction, coronary artery disease, and cerebrovascular accidents.

Insight into the pharmacodynamics of CGRP could shed light on possible risks associated with long-term blockade of CGRP. The present systematic review summarizes the current literature on reported physiological role of CGRP reflected by vascular changes following infusion of h-α-CGRP in healthy volunteers and individuals diagnosed with migraine, discuss the physiological and pathophysiological effects based on the reported adverse events (AEs), and highlights potential risks when targeting CGRP signaling pathway.

## 2. Methods

### 2.1. Data sources

An *a priori* systematic review protocol was developed. Although no protocol was registered, the full protocol can be obtained from the corresponding author upon request. The reporting of this systematic review was guided by the standards of the Preferred Reporting Items for Systematic Review and Meta-Analysis (PRISMA) Statement (14). We searched PubMed and Embase for human studies in English using intravenous infusion of h-α-CGRP prior to July 14, 2022, with no demographic specification. The search string was “(CGRP OR ‘calcitonin gene-related peptide’) AND (Infusion OR Administration)”.

### 2.2. Selection criteria, study inclusion, and data extraction

After de-duplicating, two investigators (M.M.K. and P.S.F) independently screened all studies by title and abstract and then full text to confirm eligibility for this review. The eligibility of studies was based on the PICO (population, intervention, comparison, and outcome) approach. Inclusion criteria were *in vivo* human studies published in English with a 20-minute (min) intravenous infusion of h-α-CGRP. We excluded studies evaluating medical treatment based on the induced effects of h-α-CGRP, studies using CGRP-antagonists and studies applying other types of headache-inducing substances. Reference lists of all included studies were screened manually for studies that had been missed by the initial search.

Data was extracted independently by two investigators (M.M.K. and P.S.F). Any discrepancies between two investigators were resolved by a third investigator (F.M.A). The following data was extracted for each included study: population, participants included, gender, drug, infusion-dose (μg/min), infusion-time (min), all mentioned AEs hypothesis, and outcome.

## 3. Results

**SCHEME 1:**
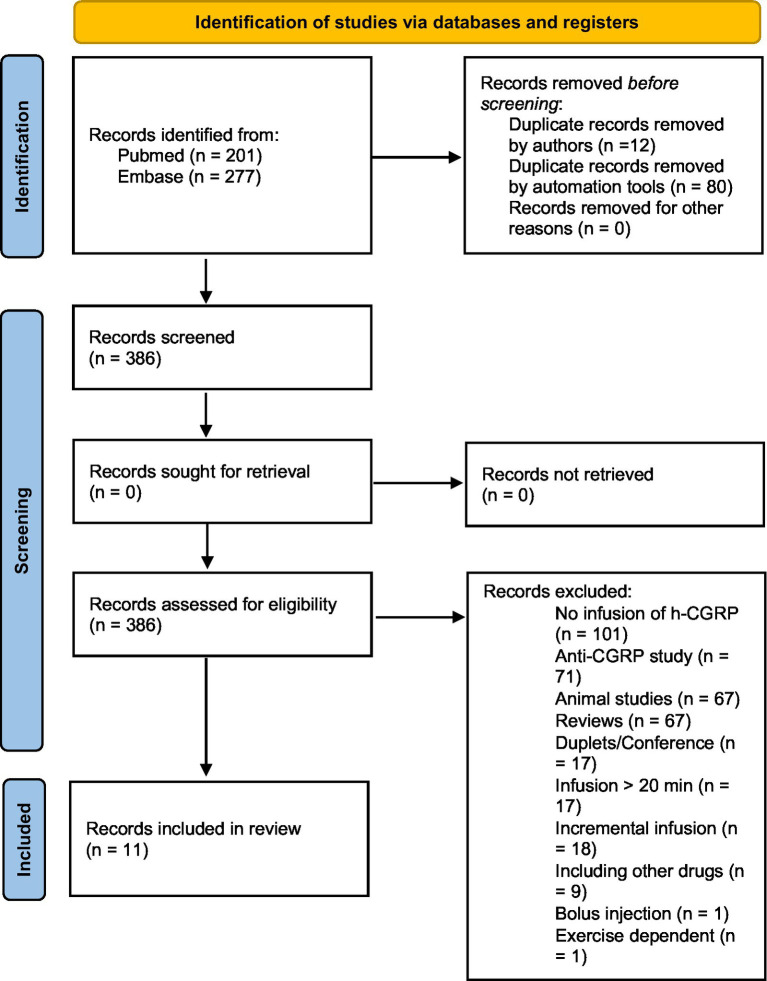
Flowchart of search protocol.

The database search identified 386 citations after removing duplicates. Through manual screening of title, abstract and full text review of relevant articles, 11 studies met the inclusion criteria and were included in Scheme 1 (11, 12, 15–23). Data following infusion of h-α-CGRP, but not placebo, was included (Tables 1–3).

**Table 1:** Population, hypothesis, and outcome.

Author (year)	Population	Study design	Hypothesis/purpose	Outcome
Lassen et al. (11, 16)*	Migraine without aura	Double-blind, placebo-controlled crossover.	2002: To elucidate the role of CGRP in known migraineurs. 2008: To investigate the cerebral hemodynamic effects of CGRP in known migraineurs.	2002: CGRP induces headache in virtually all migraineurs. 2008: CGRP appears to dilate cerebral arteries, but the effect is unlikely to be the sole mechanism of CGRP-induced headache.
Hansen et al. (15)	FHM with known mutations	Balancer, controller, single-blinded	The FHM genotype confers a CGRP hypersensitivity phenotype.	The FHM genotype does not show hypersensitivity to the CGRP-pathway.
	Healthy volunteers			
Hansen et al. (12, 23)*	Migraine with aura	Non-randomized, balanced, controlled, single-blinded	Examining the migraine inducing effect of CGRP in patients with migraine with aura.	CGRP triggers migraine in patients with migraine with aura, indicating a similar pathway for migraineurs with/without aura.
	FHM without known mutations	Non-randomized, balanced, controlled	Examining the migraine inducing effect of CGRP in FHM patients without known mutations	No difference was found between the two groups.
	Healthy volunteers	–	A control group.	–
Guo et al. (17)	Migraine without aura	Randomized, balanced, double-blinded	Migraineurs with a high family load of migraine would report more migraine attacks after CGRP infusion vs. low family load.	No statistical association of familial aggregation.
Christensen et al. (18)	Chronic and episodic migraineurs with/without aura	Double-blind, placebo-controlled crossover.	To explore a possible association between individual efficacy of anti-CGRP treatment and susceptibility of migraine induction of CGRP.	Patients with previous effect of Erenumab are highly susceptible to CGRP-induced migraine-like headache.
Visocnik et al. (19)	Healthy volunteers	Non-randomized, non-blinded	Intravenous CGRP may have a significant vascular effect on both MCA and PCA and on systemic circulation.	CGRP induces changes in cerebral and systemic circulation in healthy volunteers.
Iljazi et al. (20)	Chronic migraine with current headache	Non-randomized, single-arm, open-label study	To investigate whether CGRP infusion induces headache in patients suffering from chronic migraine, not having/having headache on the experimental day.	CM patients are hypersensitive to CGRP. The potency of CGRP is increased in chronic migraineurs with ongoing headache.
	Chronic migraine without current headache			
Coskun et al. (22)	Healthy volunteers	A randomized, double-blind, placebo-controlled, cross-over study	To investigate whether pretreatment with glibenclamide or placebo inhibits CGRP induced	Pretreatment with a non-selective K_ATP_ channel inhibitor glibenclamide did not attenuate CGRP-induced headache and hemodynamic changes in healthy volunteers.
Zupan et al. (21)	Migraine without aura	Non-randomized, non-blinded	The cerebral hemodynamic response to CGRP infusion differs between MO and MA, reflecting a different degree of trigeminovascular sensitization.	The cerebral hemodymanics differs between MO and MA, indicating a pronounced vasodilation and trigeminovascular sensitization in MA.
	Migraine with aura			

*, same population; CGRP, calcitonin gene-related peptide; FHM, familiar hemiplegic migraine; CM, chronic migraine; MO, migraine without aura; MA, migraine with aura.

**Table 2:** Headache.

Author (year)	Population	n	Age (years)	M:F	Infusion (μg/min; min)	Headache	Migraine-like headache	Aura	RM
Lassen et al. (11, 16)*	Migraine without aura	12	39.5	1:11	H-α-CGRP (2.0; 20)	9/9	3/9	-	3/9
Hansen et al. (15)	FHM with known mutations	9	38	2:7	H-α-CGRP (1.5; 20)	–	2/9	0/9	–
	Healthy volunteers	10	32	6:4	H-α-CGRP (1.5; 20)	–	1/10	0/10	–
Hansen et al. (12, 23)*	Migraine with aura	14	43	6:8	H-α-CGRP (1.5; 20)	12/14	8/14	4/14	2/14
	FHM without known mutations	11	43	3:8	H-α-CGRP (1.5; 20)	8/11	1/11	–	1/11
	Healthy volunteers	11	36	7:4	H-α-CGRP (1.5; 20)	7/11	0/11	–	1/11
Guo et al. (17)	Migraine without aura	40	45.5	4:36	H-α-CGRP (1.5; 20)	–	24/39	–	17/39
Christensen et al. (18)	Chronic and episodic migraineurs with/without aura	13	39	1:12	H-α-CGRP (1.5; 20)	–	10/13	–	10/13
Visocnik et al. (19)	Healthy volunteers	20	39	11:9	H-α-CGRP (1.5; 20)	–	–	–	–
Iljazi et al. (20)	Chronic migraine with current headache	38	43	4:34	H-α-CGRP (1.5; 20)	–	35/38	–	–
	Chronic migraine without current headache	20	43	2:18	H-α-CGRP (1.5; 20)	–	13/20	–	–
Coskun et al. (22)	Healthy volunteers	20	23	9:11	H-α-CGRP (1.5; 20)	19/20	2/20	–	1/20
Zupan et al. (21)	Migraine without aura	15	40	5:10	H-α-CGRP (1.5; 20)	11/15	–	0/15	–
	Migraine with aura	5	40	0:5		5/5		0/5	

*, same population; M, male; F, female; RM, rescue medication; CGRP, calcitonin gene-related peptide; –, not reported.

**Table 3:** Vascular effects.

Author (year)	Flushing	HB	HT	HR	MAP	V_MCA_	STA dilation	EndTidal pCO2
Lassen et al. (11, 16)*	10/10	–	10/10	+58.1% (>80 min)	−12.2% (>20 min)	−13.5% (75 min)	–	−6.8% (NR)
Hansen et al. (15)	–	–	–	Increase, not specified	–	−9.6% (>30 min)	42.8% (>120 min)	–
	–	–	–	Increase, not specified	–	−9.6% (>20 min)	42.5% (>120 min)	–
Hansen et al. (12, 23)*	13/14	8/14	13/14	+25.7% (>60 min)	−6.8% (>30 min)	–	–	–
	11/11	2/11	11/11	+29.4% (>60 min)	−6.7% (>60 min)	–	–	–
	–	–	–	+13.8% (>60 min)	−10.0% (>60 min)	–	–	–
Guo et al. (17)	39/39	21/39	37/39	–	–	–	–	–
Christensen et al. (18)	13/13	5/13	13/13	–	–	–	–	–
Visocnik et al. (19)	–	–	–	Increase, not specified	Decrease, not specified	Decrease, not specified	**–**	Decrease, not specified
Iljazi et al. (20)	57/58	45/58	56/58	+49% (>60 min)	−12.3% (>50 min)	–	–	–
Coskun et al. (22)	20/20	17/20	20/20	+30% (>120 min)	−9% (>50 min)	−21% (>30 min)	41% (>120 min)	**No change**
Coskun et al. (22)	–	–	–	+18.98% (>40 min)	+11.05% (>40 min)	−9.47% (>40 min)	**–**	−2.66% (>40 min)

*, same population; –, not specified; Yes/No, significant change; HB, heartbeat (palpitation); HT, heat; HR, heart rate; MAP, mean arterial pressure; V_MCA_, mean velocity middle cerebral artery; (min), duration; pCO2, partial pressure of CO2.

In total, 238 adults were included in the 11 studies: two studies included healthy participants exclusively (*n* = 40), four studies included participants diagnosed with migraine without aura (MO, *n* = 67) (11, 16, 17, 20), one study included participants diagnosed with migraine with aura (MA, *n* = 19), two studies included participants diagnosed with chronic migraine (CM, *n* = 71) (18, 20), two studies included participants diagnosed with familial hemiplegic migraine (FHM, *n* = 20) (15, 23). In three studies with participants with migraine, healthy adults (*n* = 21) were included as a control group (12, 15, 23). In total, 11 studies included 61 healthy participants and 177 participants diagnosed with migraine (11, 12, 15–23; Table 2).

Objective and subjective measurements on the hemodynamic effects following h-α-CGRP infusion included flushing, palpitation, warm sensation, heart rate (HR), mean arterial blood pressure (MABP), mean blood flow velocity of middle cerebral artery (mean V_MCA_) and diameter of superficial temporal artery (STA) (Table 3). Upon the start of h-α-CGRP infusion, 163 of 165 (99%) participants had flushing, 98 of 155 (63%) participants reported palpitation, and 160 of 165 (97%) participants reported warm sensation. Lassen et al. found the median time of flushing to be 70 min after the start of h-α-CGRP infusion (11, 16). HR increased with 14%–58% and MABP decreased with 7%–12%. One study found an increase of MABP with 11% after CGRP. The mean V_MCA_ was decreased with 9.5%–21% (11, 15, 16, 19, 21) and the diameter of the STA was dilated with 41%–43%. Based on the blood flow velocity decrease, Lassen et al. (16) estimated the dilation of the MCA to be 7.5%, given that the total blood flow is constant. The peak vascular changes occurred at 15–20 min after the start of h-α-CGRP infusion and lasted from 20 to >120 min.

Upon h-α-CGRP infusion, 26 of 31 (84%) of healthy participants reported headache (12, 23), three of 41 (7%) healthy participants reported migraine-like headache (12, 15, 23), and 96 of 153 (63%) participants diagnosed with migraine reported migraine-like attacks (11, 12, 15–18, 20, 21, 23).

Nausea was reported by 35 of 86 (41%) participants diagnosed with migraine in five studies (11, 12, 17, 18, 23). Aura was investigated in 3 studies conducted on participants diagnosed with FHM, MA exclusively, and MO and MA (12, 15, 21). Four of 53 (8%) participants reported aura following infusion of h-α-CGRP. All four cases of aura were observed in Hansen et al. (12). Five studies reported that 35 of 46 (76%) participants used their rescue medications to treat the induced migraine (11, 12, 17, 18, 23).

## 4. Discussion

The present study is the first to systematically review and evaluate the vascular effects following intravenous infusion of h-α-CGRP (Tables 1–3). Overall, infusion of h-α-CGRP caused no serious AEs and was well-tolerated by healthy participants and individuals diagnosed with migraine. Infusion of h-α-CGRP caused flushing, palpitation, warm sensation and dilated cerebral and extra-cerebral arteries. The peak vascular changes occurred at 15–20 min after the start of h-α-CGRP infusion and lasted 20–120 min across studies. These results reflect the short half-life of CGRP (approximately 7–30 min) (24) and its effect on G protein-coupled receptor (GPCR).

In mice, genetic deletion of CGRP or RAMP1 elevated baseline blood pressure in some (25–29) but not all studies (5, 30). Overexpression or knock-in of human RAMP1 in all (31) or solely neural tissues (32) potentiates CGRP-dependent blood pressure reduction in angiotensin II-induced hypertension. Numerous *in vivo* preclinical studies of hypertension and heart failure demonstrated an upregulation of RAMP1 and/or CGRP expression in pathological conditions, indicating a cardioprotective role of CGRP (5, 8–10). Thus, CGRP might delay the onset and development of hypertension through cardioprotective mechanisms in addition to ameliorating pressure overload-induced heart failure. Moreover, the presence of CGRP receptor blockers inhibited dilation of collateral vessels in acute ischaemic stroke, leading to an increase in infarct volume (33). Collectively, CGRP is a key physiological regulator of vascular tone with cardioprotective effects. However, studies investigating the beneficial effects of CGRP have been limited due to its short peptide half-life.

### 4.1. Targeting CGRP pathway

Trigeminovascular release of the potent vasodilatory neuropeptide CGRP has been shown to have an essential role in migraine attack initiation (34). Its involvement in migraine pathogenesis has been firmly established by a series of intervention studies, wherein patients with migraine reported migraine-like attacks upon intravenous CGRP infusion (11, 12). These findings have emphasized that CGRP pathway is a promising target and have led to development of migraine-specific therapies, including CGRP receptor antagonists (gepants: ubrogepant, zavegepant, rimegepant, and atogepant) and CGRP-pathway targeting monoclonal antibodies (eptinezumab, erenumab, fremanezumab, and galcanezumab) (35). The approval of gepants and monoclonal antibodies by the US Food and Drug Administration (FDA) and the European Medicines Agency, shifts these from administration in controlled clinical trials to the real-world setting. It is worth mentioning that the vast majority of clinical trials included women of a White ethnic background of similar ages from the USA and Europe. The fundamental question is whether findings from these trials are replicable in populations with no restricted diversity.

In the past three decades, triptans (5-HT1B/1D receptor agonists) were the gold standard acute-acting antimigraine treatments (36). Triptans, which were highly effective and well-tolerated, have revolutionized the treatment of this debilitating condition that affects millions of people throughout the world. Although the cardiovascular risk of triptans appears to be low, the use of triptans in clinical practice is contraindicated when having a history of a myocardial infarction, coronary artery disease, and cerebrovascular accidents (37, 38). Moreover, cardio-and cerebrovascular risk could be increased by chronic use of non-steroidal anti-inflammatory drugs (NSAID) (39). A novel acute therapy for migraine is lasmiditan which is a centrally-penetrant, highly selective and potent 5-hydroxytryptamine type 1F (5-HT_1F_) receptor agonist without vasoconstrictive activity (40). However, lasmiditan is associated with impaired driving performance, patients are advised not to drive a motor vehicle or operate machinery for at least 8 hours after ingestion (41). These observations emphasized the unmet need for migraine-specific therapies without cardio-and/or cerebrovascular risk.

The second generation of gepants are small-molecule CGRP receptor antagonists that are administrated orally (ubrogepant, rimegepant, and atogepant) or intranasally (zavegepant). Clinical trials showed that gepants are safe, tolerable and effective for the acute (ubrogepant, rimegepant, and zavegepant) and preventive (rimegepant and atogepant) treatment of migraine (42–45). Owing their high lipophilicity, gepants largely distribute to adipose tissues, and such fat accumulation would lead to long-lasting release of gepants into blood. In the broad and historical sense, the population response of gepants in terms of pain relief and freedom from the most bothersome migraine associated symptoms at 2 hours is less than that seen with triptans. Due to the limitations of comparing across studies, it might not be accurate to conclude that gepants have a lower efficacy compared to triptans. In a randomized, double-blind, placebo controlled, dose-ranging study with triptan as an active comparator, responses to the rimegepant and sumatriptan on pain freedom were similar (46). Clinical trials reported that ubrogepant was safe in healthy adults and in individuals with a moderate to high cardiovascular risk profile (47, 48). The safety of ubrogepant was sustained, as long-term (over at least 1 year) intermittent use did not induce clinically significant cardiac or hepatic adverse events (49). Hence, gepants might be effective in patients with comorbid cardiovascular disease, vascular risk factors and/or in patients with insufficient response to other migraine therapies. However, these studies were usually performed in otherwise healthy women of a White ethnic background with high BMIs, and were not powered to detect effect of sex, BMI, or ethnic background. These factors might in particular combinations lead to either high drug concentrations, increasing side-effect burden, or low drug concentrations, reducing drug efficacy. Therefore, prospective randomized studies including diverse patient populations with comorbid cardiovascular disease are needed. Collectively, several aspects might affect exposure and clinical response to gepants in a wide range of patients.

Monoclonal antibodies are a new antimigraine drug class, targeting either CGRP (fremanezumab, galcanezumab, and eptinezumab) or its receptor (erenumab). The last is a fully human monoclonal antibody, whereas the other three antibodies contain a sequence of the mouse genome (35). The net result of targeting CGRP or its receptor is that interaction between CGRP and its receptor can no longer occur in a similar way to the gepants. However, in the case of erenumab, CGRP is free to act on other receptors (eg, the amylin-1 receptor) (43, 50). Due to their long half-life, combined with their route of administration (intravenous or subcutaneous), interindividual variability is less expected for the antibodies compared to the gepants. Pharmacokinetic and pharmacodynamic dose-finding studies reported that CGRP-induced dermal vasodilation lasted up to 12 h in human forearm model (51), and the antibody concentrations that were needed to inhibit CGRP-induced vascular effects were considerably lower than those required for efficient antimigraine therapy (52). Possible explanations for this observation are (1) simple cephalic vasodilation is not sufficient to trigger migraine pain, (2) vascular bed in the trigeminal region is different from extra-trigeminal region with regard to the released amount of CGRP and/or the CGRP receptor density, and (3) different combination of receptors involved in CGRP-induced vascular response in the trigeminal region. The last explanation is further supported by the finding that patients with migraine without aura reported migraine attacks upon intravenous infusion of amylin (53). The relative contribution of these different receptors is yet to be elucidated. One important difference after activation is that the canonical CGRP receptor undergoes internalization (ie, it brought into the cell, but can still signal), while internalization does not seem to occur with the amylin-1 receptor. Considering the role of CGRP in cardiovascular health (54, 55) and because the cardiovascular risk of long-term use of drugs targeting CGRP pathway is unknown, the general recommendation is that the new drugs should not be used in people at high risk for ischaemic events (56–58). Clinical trials assessing the impact of anti-CGRP therapies on blood pressure (BP) reported conflicting results. Erenumab and fremanezumab were found to increase the mean systolic and diastolic BP, and some patients required antihypertensive treatment (59, 60). However, Dodick and collogues (61) found no increased risk of hypertension in patients with migraine who received erenumab in clinical trials and in the postmarketing setting and concluded that additional data are needed to fully characterize the extent to which hypertension is a risk associated with targeting CGRP pathway. In a safety follow-up study (62), 5 years of erenumab treatment had no meaningful changes in mean systolic/diastolic blood pressure or heart rate compared to baseline. Large-scale follow-up clinical trials with a wide range of age groups will clarify whether long-term CGRP blockade leads to hypertension-related side effects.

## 5. Limitations and strengths

The present study is the first to systematically review and evaluate the vascular effects following intravenous infusion of h-α-CGRP. We acknowledge that only including studies with a 20-min intravenous infusion of h-α-CGRP is a limitation. The reason behind this inclusion criteria is to capture homogeneous vascular data. We included all studies that fulfilled the predefined ex-and inclusion criteria, but the majority of the included studies were performed in our center. Despite the limitation that several studies did not assess the effect of α-CGRP on V_MCA_ and the diameter of the STA (Table 2), the consensus finding is that α-CGRP decreased mean V_MCA_ (with ~10%) and increased the diameter of the STA (with ~40%).

## 6. Conclusion

Infusion of h-α-CGRP caused flushing, palpitation, warm sensation and dilated cerebral and extra-cerebral arteries. The peak vascular changes occurred at 15–20 min after the start of h-α-CGRP infusion and lasted 20–120 min across studies. Thus, CGRP is a key physiological regulator of vascular tone, and intravenous infusion of h-α-CGRP caused a universal vasodilation and was well-tolerated with no serious AEs. Whether long-term blockade of CGRP in patients with migraine could cause cardiovascular complications or ischaemic events is yet to be clarified.

## Author contributions

MA-K and FA initiated and contributed to study design, data acquisition, data processing, analysis, and interpretation, and drafting and revision of the paper. VK and PF contributed to data acquisition, data processing, analysis, interpretation, and drafting of the paper. All authors approved the submitted version.

## Conflict of interest

The authors declare that the research was conducted in the absence of any commercial or financial relationships that could be construed as a potential conflict of interest.

## Publisher’s note

All claims expressed in this article are solely those of the authors and do not necessarily represent those of their affiliated organizations, or those of the publisher, the editors and the reviewers. Any product that may be evaluated in this article, or claim that may be made by its manufacturer, is not guaranteed or endorsed by the publisher.
